# Genome sequence and annotation of *Streptomyces tendae* UTMC 3329, acid and alkaline tolerant actinobacterium

**DOI:** 10.18502/ijm.v12i4.3939

**Published:** 2020-08

**Authors:** Lida Eftekharivash, Javad Hamedi

**Affiliations:** 1 Department of Microbial Biotechnology, School of Biology and Center of Excellence in Phylogeny of Living Organisms, College of Sciences, University of Tehran, Tehran, Iran; 2 Microbial Technology and Products Research Center, University of Tehran, Tehran, Iran

**Keywords:** Actinobacteria, Acid-tolerant, Alkaline-tolerant, Genome annotation, Genome sequencing, Genome mining, *Streptomyces tendae*

## Abstract

**Background and Objectives:**

*Streptomyces tendae* is one of the most prolific actinobacteria with a wide range of biotechnological applications. Genomic data can help in better understanding and exploration of important microorganisms, however, there is a few genomic information available for this species.

**Materials and Methods:**

Molecular identification, pH and salt tolerance of an actinobacterium, designated *Streptomyces tendae* UTMC 3329, isolated from a tea field soil were done. Also, genomic DNA was extracted and sequenced using Illumina platform with MPS (massively parallel sequencing) Illumina technology. Gene annotation and bioinformatic analysis were done using appropriate software and servers.

**Results:**

The draft genome is ∼8.7 megabase pairs, containing 7557 predicted coding sequences. The strain was able to grow at pH 5–12 and 0–10% NaCl. The maximum growth rate of the bacterium was obtained at pH 7. The gene clusters involved in central carbon metabolism, phosphate regulation, transport system, stress responses were revealed. It was shown the presence of gene clusters of polyketides, ribosomally and non-ribosomally synthesized peptides. Various genes were found in xenobiotic degradation pathways and heavy metal resistance.

**Conclusion:**

The current genomic information which reveals biological features, as well as the biotechnological potential of this acid and alkaline tolerant actinobacterium, can be implemented for further research on the species.

## INTRODUCTION

Actinobacteria are one of the most important bacterial members that are wide-spread in marine and terrestrial habitats. These bacteria drive many critical biogeographically important processes such as playing roles in carbon and nitrogen cycles. Members of the *Streptomyces* genus, as the most dominant actinobacteria (1, 2) are known to be involved in carbon recycling and breaking down complex biological polymers due to their ability to produce various hydrolytic exoenzymes. Over the years, continuous efforts of isolation and screening have been led to the discovery of many commercial drugs with the actinobacterial origin which many of them (over 150,000) are derived from *Streptomyces* genus (3–5). These biocompounds exhibit noteworthy therapeutic activities such as antiviral, antibacterial, antifungal, anticancer, antioxidative and neuroprotective, cytotoxic, cytostatic, anti-inflammatory, anti-parasitic, anti-malaria, antiviral and anti-angiogenesis activities. Because of the significant value of *Streptomyces*, these filamentous bacteria remain a hot spot for research on their application in white, red, blue and green biotechnology sections (6). The unknown potential of *Streptomyces* is being revealed by whole-genome sequencing through identification and expression of low expressed, silent, or cryptic biosynthetic gene clusters (7).

At the time of writing the current report, whole-genome sequences for 216 *Streptomyces* species have been sequenced, among which 184 genomes belong to corresponded type species. Moreover, 32 non-type species of *Streptomyces* have been sequenced on which no report from their corresponded type species is available (8). Meanwhile, during doing this research, two genomes were submitted in NCBI as *Streptomyces tendae*, including strain 139 (16 Sep 2019) and strain VITAKN (25 Jan 2020). Their accession numbers were PRJNA565833 and PRJNA600621, respectively. There is no article for the strain 139, however, it has 99.52% similarity to ATCC 19812 (T) according to the analysis of the 16SrRNA gene obtained from the genomic data submitted in NCBI. The strain VITAKN has 99.8% similarity to ATCC 19812 (T) as reported by Ishaque et al. (9), however, there is no sequences for the 16Sr-RNA gene in the whole genome sequences submitted in NCBI for the strain VITAKN in NCBI (PRJNA600621).

*S. tendae* is one of the most prolific members of *Streptomyces* genus. It was firstly isolated from soil of Tende, France (10). By the time of this writing, 24 strains of *S. tendae* were reported to be capable of synthesizing various biocompounds discovered through different phenotypical studies. No pathogenesis has been recorded on *S. tendae* on animal, plant and human making this species to be categorized as risk group I of microorganisms. Considering the biotechnological impact of *S. tendae* and lack of sufficient genomic information for this species, in this report, genome information of a strain of this actinobacterium isolated from a tea field (11) was sequenced and submitted in NCBI (PRJNA5077186, 03 DEC2018). Based on genome annotation and analysis, biological and biotechnological features such as metabolite production potentials and physiological features were revealed as discussed hereafter.

## MATERIALS AND METHODS

### Actinobacteria strain.

An actinobacterium designated *S. tendae* UTMC 3329 was obtained from the University of Tehran Microorganisms Collection. It has been isolated in a screening program, from a tea field soil (Gerd-Korf village (37.0756040N, 50.0148287E), Lahijan, Iran) with pH 5.5 (11).

### pH and salt tolerance profiles of the strain.

The strain was cultured in ISP-2 broth at pH 1–14 or various concentrations of NaCl (0–12% w/v) and incubated at 28°C, 14 days (12). Also, appropriate concentrations of spores (∼10^7^–10^8^/ml) were added into the 2000 ml Erlenmeyer containing 300 ml ISP-2 broth various pH (5–7) and incubated at 28°C, 180 rpm, 48 h. For measuring the growth, the wet biomass was calculated as the ratio of the packed cell weight to the wet weight of the culture medium, after centrifuging the broth samples at 10,000 g, 20 min. To study morphology, each broth sample was Gram-stained and 30 fields of view were captured randomly by a light microscope, and the predominant morphology was determined and reported (13).

### Molecular identification.

The biomass of the strain was prepared and its DNA was extracted using DNA extraction Kit (Pooya Gene Azma Co., Tehran, Iran). The 16S rRNA gene was amplified using 9F (5′-AAGAGTTTGATCATGGCTCAG-3′) and 1541R (5′-AAGGAGGTGATCCAGCC-3′) primers (14). The PCR products were sequenced using ABI 3730XL DNA analyzer (Applied Biosystems) and compared with that of other validated species in EzBioCloud database using 16S-based ID service from EZBio-Cloud (8). Rather than sequencing of the 16S rRNA gene by Sanger method, its sequence was extracted from the sequences genome based on its annotation which had been performed by RAST (15) and was analyzed by EZBioCloud (8). Moreover, a phylogenetic tree for *S. tendae* and its type strain neighbors was constructed using MEGA X (16).

### Genome sequencing.

Genomic DNA (gDNA) was extracted and whole-genome sequencing was performed on the Illumina platform with MPS (massively parallel sequencing) Illumina platform. A-tailed, ligated to paired-end adaptors and PCR amplified with a 500 bp insert and a mate-pair library with an insert size of 5 kb were used for the library construction at the Novogene Co.

Illumina PCR adapter reads and low quality reads from the paired-end and mate-pair library were filtered and all good-quality paired reads were assembled using the SOAPdenovo (17) resulted in 83 scaffolds of >512 bp, a length of 8, 653, 184 bp and a GC content of 72.44%. read quality was assessed using FastQC (18). The filter reads were handled by the next step of the gap-closing. GeneMarkS program to retrieve the related coding genes (19).

### Gene annotation and bioinformatics analysis.

The interspersed repetitive sequences, tandem repeats, transfer RNA and ribosome RNA (rRNA) genes were predicted using the RepeatMasker (20), TRF (Tandem repeats finder) (21), tRNAscan-SE (22) and RNAmmer (23), respectively. Small RNAs (sRNA) were predicted by BLAST against the Rfam database (24). CRISPRFinder was also used for the CRISPR identification (25).

Using RAST (26) and COG (Clusters of Orthologous Groups) (27), the functional annotation of genes was elucidated. To further analyze, the genome-based metabolic potentials as well as comparative genome analysis of the current strain, the RAST/Model SEED framework and OrthoFinder (28) were implemented, respectively. OrthoVenn web server (29) was also used by its ClusterVenn tool to visualize genome comparison results gained by OrthoFinder.

## RESULTS

### Microbiological information.

**Fig. 1.**
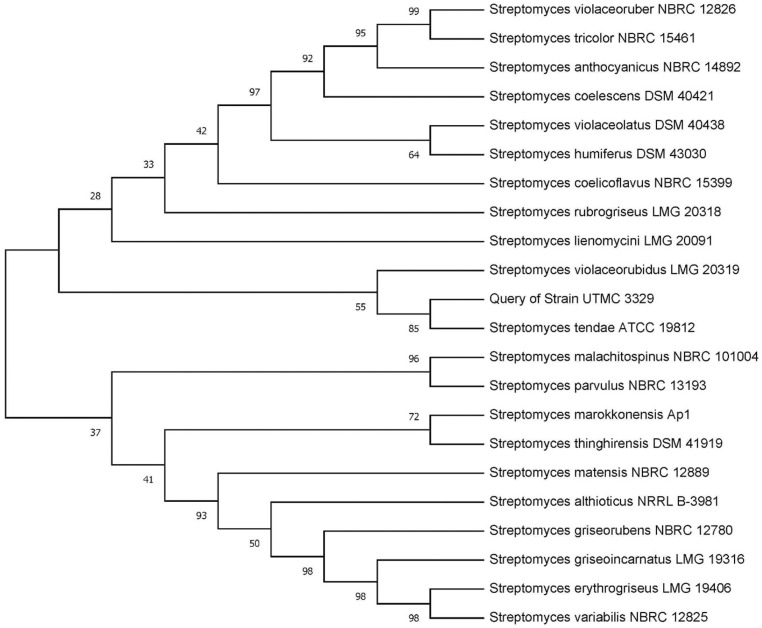
Phylogenetic tree of the strain of *Streptomyces tendae* UTMC 3329 and other relative type strains of the genus *Streptomyces* inferred from 16S rRNA gene sequences under the neighbor-joining method. Bootstrap values (>50%) are indicated at the relevant branching points.

The strain UTMC 3329 produced well-developed substrate mycelium and an aerial mycelium with short, compact, spiral spore chains. Based on 16S rRNA gene sequences, and the data obtained from the house-keeping genes sequences genome annotated by RAST, the strain UTMC 3329 was 100% related to *Streptomyces tendae* ATCC 19812T (Fig. 1).

**Fig. 2.**
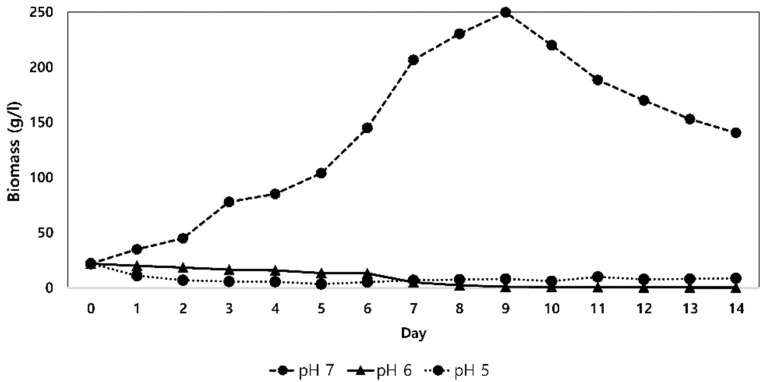
Effects of acidity on the growth of *S. tendae* UTMC 3329.

The strain was able to grow at pH 5–12 but did not grow at pH 4 or 13. Moreover, the experimental results showed that *S. tendae* can grow in 0–10% of NaCl. The results of the growth curves of the bacterium at various pH (5–7) are shown in Fig. 2. Maximum growth rate (μ) at pH 5, 6, and 7 were, −0.13, −0.26, and 0.24, respectively.

**Fig. 3.**
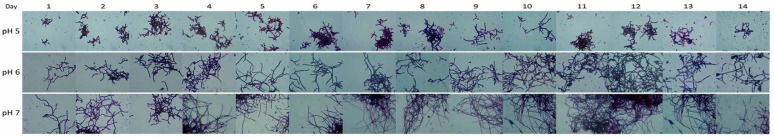
Effects of acidity on the morphology of *S. tendae* UTMC 3329.

Morphology of the strain at various pH values is also shown in Fig. 3. Maximum biomass concentration was obtained in the medium with pH 7 and the minimum biomass concentration was seen in the medium with pH 5. The life-time and morphology of *S. tendae* were affected by the pH of the medium. In the medium with pH 6, hyphae were remained in a vegetative form after 12 days, while in the medium with pH 5, spores were formed and hyphae were lysed. In the medium with pH 7, hyphae were longer and slenderer, while was less branched than that of the medium with pH 6.

### Genomic information.

The overall properties of genetic information of *Streptomyces tendae* UTMC 3329 are summarized in Table 1. This whole-genome shotgun (WGS) project has been deposited at Gen-Bank under the accession BioProject: PRJNA224116; BioSample: SAMN10485464.

**Table 1.** General genomic features of the draft sequence of *Streptomyces tendae* UTMC 3329.

**Feature**	**Value**
Genome size (bp)	8653184 bp
DNA coding region (bp)	7549236 bp
% of Genome (Genes)	87.24
GC content (%)	72.44
Total genes	7,635
RNA genes	78
rRNA operons	5
tRNA genes	65
sRNA	8
Protein-coding genes (CDSs)	7557
Psuedogenes	1
Genes assigned to COGs	6711

### Screening the genome based on the COG functional categories.

**Fig. 4.**
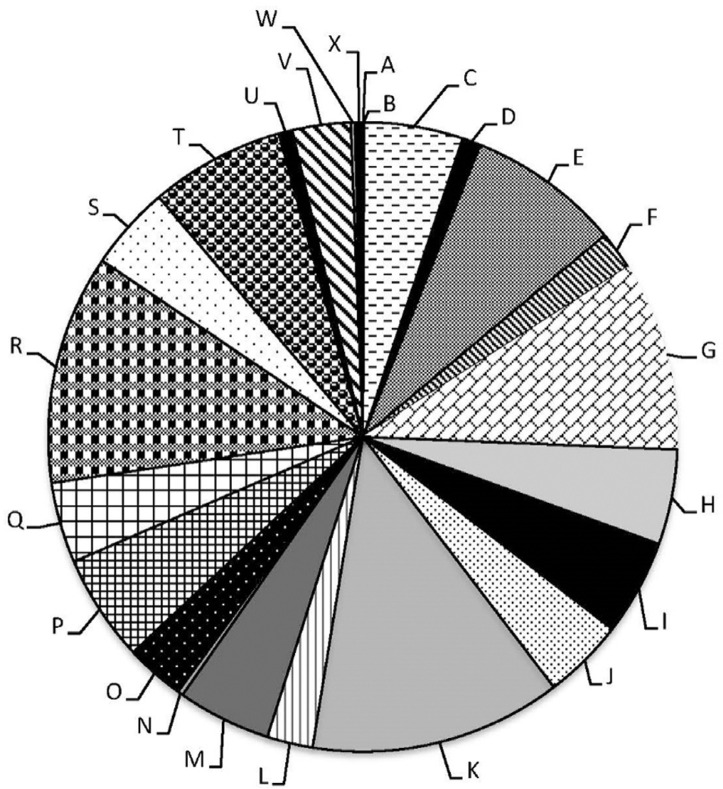
Subsystems category distribution of *Streptomyces tendae* UTMC 3329 genes based on COG. (The 25 general COG functional categories are shown as A: RNA processing and modification; B: Chromatin structure and dynamics; C: Energy production and conversion; D: Cell cycle control, cell division, chromosome partitioning; E: Amino acid transport and metabolism; F: Nucleotide transport and metabolism; G: Carbohydrate transport and metabolism; H: Coenzyme transport and metabolism; I: Lipid transport and metabolism; J: Translation, ribosomal structure and biogenesis; K: Transcription; L: Replication, recombination and repair; M: Cell wall/membrane/envelope biogenesis; N: Cell motility; O: Posttranslational modification, protein turnover, chaperones; P: Inorganic ion transport and metabolism; Q: Secondary metabolites biosynthesis, transport and catabolism; R: General function prediction only; S: Function unknown; T: Signal transduction mechanisms; U: Intracellular trafficking, secretion, and vesicular transport; V: Defense mechanisms; W: Extracellular structures; X: Mobilome: prophages, transposons).

The results of the genome classification of the sequenced genome according to COG functional categories are shown in Fig. 4. Among 7635 total genes, 278 genes are involved in the biosynthesis of secondary metabolites. It is important to note that 924 genes could not be classified in any COG categories.

### Secondary metabolite.

The secondary metabolite potential of *S. tendae* according to BAGEL4 (30) and antiSMASH 4.0 (31) tools, are found. Based on BAGEL4, five novel lanthipeptides were identified: one class I lanthipeptide and four class III lanthipeptides. antiSMASH resulted in the prediction of some metabolites, including, coelichelin, alkylresorcinol, isorenieratene, albaflavenone, ectoine, coelibactin, melanin and desferrioxamine with 100% sequence similarity to the most similar known cluster.

### Biodegradation.

Analysis of the genome annotation using KEGG mapping of the metabolic pathways of *S. tendae* has shown that ∼3% of its annotated genes belong to xenobiotic degradation pathways. Accordingly, multiple genes encoding dioxygenase and decarboxylating dehydrogenases, ring cleavage reactions and monooxygenases were shown as high-scored annotations mainly for benzoate, aminobenzoate and naphthalene degradation. The xenobiotic degradation strategies of *S. tendae* include using them for energy supply (i.e. toluene, xylene) and degradation to less toxic derivatives (i.e. styrene).

### Metabolic features.

Based on RAST annotation, 7903 coding regions (CDS) were predicted of which near 77% was predicted as with specific functions while the rest being assumed as hypothetical proteins. Based on ModelSEED, complete Embden–Meyerhof pathway, glycolysis, oxidative and non-oxidative pentose phosphate pathway (PPP) and TCA cycle were seen in *S. tendae* and no difference to other members of the genus was observed. However, the genes of the Entner–Doudoroff pathway (ED) were not found.

### Phosphate regulation.

Since phosphate regulation has been shown to play important roles in antibiotics and other secondary metabolites production in *Streptomyces* spp. (32–34), its analysis can give useful insights especially in metabolic engineering of secondary metabolite production. The orthologs of the PhoR-PhoP genes, the two-components phosphate regulatory system in *S. tendae* were found. Nearly, similar or orthologs of all Pho binding proteins shown in *S. coelicolor* (35) are present in *S. tendae.* Accordingly, phosphate control in *S. tendae* was shown to interact with other regulators such as GlnR or AfsR, which further ensures the equilibrium of using different nutrients leading to optimal growth/secondary metabolite production.

### Global and local regulation.

The annotation also represents a strong emphasis on regulation, having 716 proteins predicted to have global and local regulatory functions. Eighty-seven sigma factors (global regulators) were found which would direct selective gene transcription.

### Stress response.

Sixty-seven genes were annotated as role players in stress response in *S. tendae*, including 31 stress-specific sigma factors (Table 2). *S. tendae* genome annotation shows three copies for a glycerol uptake facilitator protein and an outer membrane protein A for osmoregulation. Moreover, genes for ectoine and hydroxyectoine accumulation were presented in *S. tendae* genome and the complete ectoines biosynthesis pathway was annotated in *S. tendae*.

**Table 2.** Stress response categories and gene copies of *S. tendae* in comparison with its close strains, including, *Streptomyces coelicolor* (SCO), *Streptomyces avermitilis* (SAV), *Streptomyces griseus* (SGR), *Streptomyces scabei* (SGR), *Streptomyces tendae* (STD).

**Main category**	**Subcategory**	**SCO**	**SAV**	**SGR**	**SSC**	**STD**
Osmotic stress	Osmoregulation	3	3	3	3	4
	Choline and betaine uptake and betaine biosynthesis	8	10	10	12	8
Oxidative stress	Oxidative stress	9	11	8	10	11
	Glutathione: Biosynthesis and gamma-glutamyl cycle	4	3	2	4	4
	Glutathione: Non redox reactions	2	1	2	1	0
	Rubrerythrin	0	0	2	0	0
	Glutathione: Redox cycle	1	1	1	1	1
	Glutathionylspermidine and Trypanothione	0	1	0	1	0
Detoxification	Uptake of selenate and selenite	4	4	1	2	1
	pathway of formaldehyde detoxification	0	0	0	1	0
Miscellaneous	Sigma B: stress response regulation	33	32	18	31	31
	Dimethylarginine metabolism	4	4	4	5	4
	Bacterial hemoglobins	2	2	1	1	2
	Carbon starvation	0	0	1	1	0
	Periplasmic stress response	1	1	1	0	1

### Transport systems.

Transport reactions are also accounted for more than 7% (613 proteins) of the CDSs, most of which are ABC permeases and ATP-binding proteins responsible for the transport of nutrients. *Streptomyces*, as a saprophytic bacterium, requires to secrete large numbers of proteins majorly in order to capture nutritional needs. *S. tendae* genome encodes for more than 520 secreting proteins predicted by SignalP-5.0 web server which finds signal peptides in a protein sequence, SecretomeP 2.0 which not signal peptide triggered protein secretion targets as well as TatP 1.0. Based on the results, *S. tendae* genome comprises more than 7% of its proteins to be secretory which shows the great importance and urge of efficient protein secretory mechanisms. Based on RAST annotation, *S. tendae*, all standard and essential Sec components such as SecY, SecE, the auxiliary SecG, SecD and SecF are present. All other Sec system genes are also present in the genome such as the product of *secA* which encodes motor protein generating ATP needed for secretion as well as YajC which is shown in other bacteria to form a heterotrimeric complex with SecD and SecF and positively interact with the essential SecYEG in protein secretion (36).

Unlike Sec system, Tat system is not widely distributed in prokaryotes. Another difference is that this system helps in the secretion of pre-folded proteins. Tat pathway needs TatABC which is present in *S. tendae* along with a second copy of *tatA* which is a common feature in bacteria possessing Tat secretory system. Other putative protein secretory-associated genes were also annotated in the genome of *S. tendae* such as *aidA* which is used in a Type V secretory system as well as *tadZ* and *tadC* and are predicted to be used for the secretion of adhesins. No ESX-1/type VII secretion pathway was annotated in the genome of *S. tendae.*

## DISCUSSION

Several reports have shown the crucial impact of whole genome sequencing and genome mining to reveal the potentials of microorganisms. However, there are a few genome data for *Streptomyces tendae*. The current study found that *S. tendae* UTMC 3329 could be considered as a source of bioactive compounds and xenobiotic degradation. *S. tendae* UTMC 3329 is a neutrophil bacterium and similar to *Enterococcus faecalis* (37) their optimum growth was seen in pH 7. However, they can tolerate acidic and alkaline conditions.

A comparison of the genetic information of *Streptomyces tendae* UTMC 3329 with those of its close strains confirms that they use EMP, TCA and PPP as central metabolism pathways. Although, some actinobacteria e.g. *Nanomuraea* sp. ATCC 39272 (38), *Streptomyces tenebrarius* (39) and *Mycobacterium smegmatis* (40) metabolize glucose via the ED, however, the most relative species to *S. tendae*, including *Streptomyces coelicolor* and *Streptomyces griseous* have not ED genes (41). The pyruvate produced by glycolysis is shown to be decarboxylated to yield acetyl-Co A by pyruvate dehydrogenase complex and in the final step, acetate is produced by acetyl-CoA synthetase and the production of ATP alongside. The presence of alcohol dehydrogenases suggests that ethanol could be produced as a fermentation product. As common to many actinobacterial genomes, the presence of multiple central carbon metabolism genes’ copies was observed in the genome of *S. tendae*, which encode similar or homologous gene products. Although the core metabolic genes are majorly organized in operons, multiple copies of central metabolic genes are scattered in the chromosome (core and arms) without a tight clustering (42). This will make a challenge especially in analyzing the metabolic network of *Streptomyces* using metabolic fluxome or gene expression data and the *S. tendae* not only obeys the same pattern but also possesses more gene copies for Pdh, Sdh and Fbpase. This is mainly due to the different possible regulatory effects of same functioning genes that may happen based on different regulatory sequences upstream of the genes or operons.

**Fig. 5.**
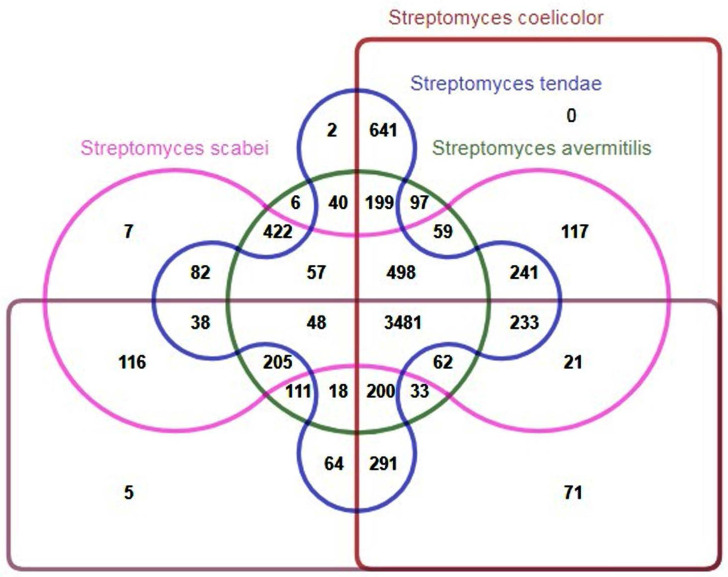
Edward’s style Venn diagram visualizing orthogroups identified by OrthoFinder among the *S. tendae* and related strains.

Genomic comparison of genes in *S. tendae* with its close relatives, including *Streptomyces coelicolor* A3 (2), *Streptomyces avermitilis* MA-4680, *Streptomyces scabiei* 87.22 and *Streptomyces griseus* subsp. *griseus* NBRC 13350 using OrthoFinder algorithm, was shown that 7465 orthogroups were detected with the maximum and minimum of 79 and 2 members in an orthogroup, respectively. There have been also unassigned genes for each strain meaning that they could not be placed in any of the orthogroups denoting that they are strain-specific genes. In the case of *S. tendae*, 805 unassigned genes were predicted as unique genes specific to this strain (supplementary file –Sheet 2). About 73% of these unassigned genes were annotated to be hypothetical and therefore they should be further characterized to unravel unique features of *S. tendae*. Others with assigned functions include both metabolic and regulatory genes. There were also two orthogroups predicted which only comprised of members both from *S. tendae* and therefore were also regarded as strain-specific genes (Fig. 5).

The current study found that the *S. tendae* has 28% global regulators (sigma factors) than that of its relative, *Streptomyces coelicolor* A3 (2) (43), keeping in mind that their genome sizes are closely similar. Production of general stress response proteins is a nonspecific response of bacteria. *Streptomyces* possess multiple types of stress-specific sigma factors that most combinations of them are involved in stress responses, adaptation to energy limitation, and development (44). Presence of 31 stress-specific sigma factors, complete ectoines biosynthesis pathway and two related heterocyclic amino acids (45), that responsible for osmotic, heat, cold, draught and pH stresses (46), show the potential of *S. tendae* in withstand in high levels of stresses.

Another important finding was that by whole genome sequencing and genome mining biological and biotechnological potentials of *S. tendae* were revealed. It was shown the presence of gene clusters of polyketides, ribosomally and non-ribosomally synthesized peptides, ectoine, and xenobiotic degradation pathways and heavy metal resistance.

Further studies on the xenobiotic biodegradation potentials of *S. tendae* UTMC 3329 might be valuable as it can be a candidate to be used in bioremediation procedures. Moreover, genes responsible for the biosynthesis of siderophores such as enterochelins have shown to be present in the genome of this bacterium which is a known characteristic of other cadmium-resistant strains of this species (47). There is no experimentally verified xenobiotic degradation reported in the species, however, *S. tendae* F4 is reported to produce siderophores useful in siderophores mediate reduced and increased uptake of cadmium (48). Generally, many members of the *Streptomyces* genus are well known to produce siderophores. Coelichelin, an iron chelator whose BGC was mined in the genome of *S. tendae* UTMC 3329, is first discovered in *S. coelicolor* (49). *S. ambofaciens, S. lividans* and *S. viridosporus*, are also known to produce different types of siderophores (49). The xenobiotic degradation and siderophore production potentials together with the presence of genes having roles in some plant hormone biosynthesis pathways such as zeatin also can denote the advantage of this strain as an endophyte and its enrolment in metabolic enrichment of soil microbial communities, study of which would result in valuable information.

Generally, having genomic-scale knowledge on the potential of secondary metabolism in *S. tendae* UTMC 3329 will greatly simplify the further characterization of bioactive compounds with a wide range of activities. Moreover, the sequenced genome improves our understanding of this organism in terms of antibiotic production and other biotechnologically-relevant features such as the bioremediation potential of this strain.
